# Digital Health Literacy in Elective Open-Heart Surgery Patients: Cross-Sectional Study

**DOI:** 10.2196/83454

**Published:** 2026-02-27

**Authors:** Rikke Daugaard, Thomas Maribo, Britt Borregaard, Ditte Sommerlund Skydt, Kristina Hindhede Bech, Kjersti Alexandra Skovli, Ivy Susanne Modrau

**Affiliations:** 1 Department of Clinical Medicine, Faculty of Health, Aarhus University, Vennelyst Boulevard 4, Aarhus, 8000, Denmark, 45 29769462; 2 Department of Cardiothoracic and Vascular Surgery, Aarhus University Hospital, Aarhus North, Denmark; 3 Department of Public Health, Faculty of Health, Aarhus University, Aarhus, Denmark; 4 DEFACTUM, Corporate Quality, Central Denmark Region, Aarhus, Denmark; 5 Department of Cardiothoracic and Vascular Surgery, Odense University Hospital, Odense, Denmark; 6 Department of Clinical Research, University of Southern Denmark, Odense, Denmark; 7 Department of Cardiology, Odense University Hospital, Odense, Denmark; 8 Esbjerg Municipal Health Centres, Esbjerg Municipality, Esbjerg, Denmark; 9 Department of Cardiothoracic Surgery, Aalborg University Hospital, Aalborg, Denmark; 10 Department of Clinical Medicine, Faculty of Medicine, University of Aalborg, Aalborg, Denmark

**Keywords:** cardiovascular diseases, thoracic surgery, elective surgical procedures, health literacy, telemedicine, computer literacy, health communication, sociodemographic factors, surveys, questionnaires

## Abstract

**Background:**

Digital health solutions play a key role in health care, but their safe and effective use depends on patients’ digital health literacy. While digital health solutions are beneficial for patients with cardiac disease, disparities in digital health literacy may limit access, particularly for patients undergoing cardiac surgery with complex care and psychological challenges. Unaddressed, these disparities could exacerbate inequalities in accessing beneficial digital services. Denmark’s advanced digital health care system provides a unique context to evaluate digital health literacy.

**Objective:**

This study aimed to assess digital health literacy levels in patients scheduled for elective open-heart surgery and examine associations with sociodemographic factors and concurrent health issues.

**Methods:**

We conducted a cross-sectional survey of consecutive patients scheduled for elective open-heart surgery at 3 university hospitals covering approximately two-thirds of Denmark’s population. Patients with cognitive impairment or language barriers preventing completion of the questionnaire were excluded. The questionnaire was administered in paper form by medical staff during preoperative consultations. Digital health literacy was assessed using the validated 8-item eHealth Literacy Scale (eHEALS; range 8‐40), along with 2 additional questions from the validated Danish version assessing the perceived importance and usefulness of online health information. Sociodemographic data collected included age, gender, cohabitation status, social support for technology use, educational level, and number of concurrent health issues. Descriptive and comparative analyses examined associations between eHEALS scores and sociodemographic variables and health issues. Exploratory subscale analyses evaluated the 3 eHEALS domains—awareness, skills, and ability to evaluate online health information—to identify areas in which patients may require additional support.

**Results:**

Of 576 eligible patients, 313 (54.3%) completed the survey between February 2024 and July 2024. Response rates varied across sites: 71.1% (133/187), 58.5% (134/229), and 28.8% (46/160) in sites 1, 2, and 3, respectively. Nonresponse was primarily due to logistical challenges during preoperative consultations, with only a few patients excluded because of cognitive impairment, language barriers, or refusal. The median eHEALS score was 30 (IQR 27‐32), indicating generally high digital health literacy scores (cutoff score ≥26). Scores were negatively correlated with age (Spearman ρ=–0.18; *P*=.002) and positively associated with educational level (Kruskal-Wallis test: *χ*^2^_2_=17.0; *P*<.001). No substantial associations were observed for gender, cohabitation status, social support for technology use, or number of concurrent health issues. Exploratory subscale analyses suggested that patients felt least confident in evaluating the quality and relevance of online information, highlighting a potential focus for tailored support.

**Conclusions:**

Patients scheduled for elective open-heart surgery generally reported high digital health literacy levels, but challenges remain in critically appraising digital health information. Younger age and higher educational levels were associated with higher self-reported digital health literacy, but the association was modest. This underscores the need for individual assessment to identify patients who may benefit from tailored support.

## Introduction

Digital health has become an essential component of modern health care delivery. Over the past decade, digital health solutions and telemedicine have steadily evolved, transforming the way in which health care is accessed and delivered. The COVID-19 pandemic significantly accelerated this development as physical consultations were limited and the need for remote solutions intensified [1,2]. Today, digital health forms an integral part of routine health care across a range of clinical settings.

These solutions are particularly valuable in outpatient cardiac rehabilitation. They can help overcome time and travel barriers during structured programs (phase 2) and support long-term recovery and lifestyle maintenance (phase 3) [3]. Digital health literacy, defined as the ability to seek, find, understand, and appraise digital health information [4], is essential when implementing digital health solutions as low digital health literacy is associated with reduced awareness and use of digital services [5]. Moreover, addressing patients’ digital health literacy may be important to ensure more inclusive implementation of these solutions, enabling broader engagement and reducing inequities [6]. Digital health interventions have been associated with improvements in health-related quality of life, as measured using validated instruments such as the HeartQoL questionnaire in cardiac populations, and with increased physical activity in patients with chronic conditions [7,8]. A recent systematic review in community-dwelling older adults reported relatively low digital health literacy as measured using the eHealth Literacy Scale (eHEALS) instrument [9]. Lower socioeconomic status and self-reported lack of digital skills have also been reported to affect digital health literacy negatively [10]. This may contribute to health disparities if health care systems do not account for patients’ digital health literacy when designing and implementing digital solutions. Consequently, patients with lower digital health literacy may face unequal opportunities to enhance their health and well-being [11]. Therefore, it is important to investigate whether sociodemographic factors are associated with digital health literacy in patients with cardiac disease, as identifying such associations could inform targeted strategies to support effective engagement with digital health interventions.

In this study, we aimed to assess digital health literacy levels in patients scheduled for open-heart surgery and examine differences across sociodemographic factors and concurrent health issues.

## Methods

### Design, Study Setting, Population, and Data Collection

This cross-sectional survey collected data via a questionnaire administered at Aarhus University Hospital, Aalborg University Hospital, and Odense University Hospital, covering 3 regions that together represent approximately two-thirds of the Danish population. Adult patients scheduled for elective open-heart surgery between February 8, 2024, and July 5, 2024, were invited to participate. Nurses or medical secretaries distributed the questionnaire in paper form to eligible patients during their preoperative consultation and collected it anonymously the day before surgery. Inclusion criteria were patients aged ≥18 years scheduled for elective open-heart surgery. Patients unable to read or understand Danish or deemed by staff to be cognitively unable to complete the questionnaire were excluded. The responsible staff members assessed whether patients were suitable to participate.

The reporting of this study follows the STROBE (Strengthening the Reporting of Observational Studies in Epidemiology) guidelines [12].

### Questionnaire Measures: Digital Health Literacy, Sociodemographics, and Data on Health Issues

The questionnaire consisted of 2 sections: one assessing patients’ self-reported digital health literacy and the other gathering their sociodemographic information and data on health issues.

We used the eHEALS to assess self-reported digital health literacy [13]. The eHEALS has been validated in multiple languages, including Danish, and applied across various demographic groups [14]. The eHEALS consists of 8 items that assess the patient’s self-perceived ability to access, evaluate, and apply health information found on the internet [15]. Respondents rate each item on a 5-point Likert scale ranging from “strongly disagree” (1 point) to “strongly agree” (5 points). Scores for each item are summed, yielding a total score of 8 to 40 points. A higher score indicates a higher level of digital health literacy, with ≥26 considered the cutoff between high and low digital health literacy levels [16-18]. Two supplementary items included in the validated Danish eHEALS assessed patients’ perceived importance and usefulness of digital health (not included in the total score) to provide contextual information on attitudes toward digital health. Furthermore, we analyzed the digital health literacy assessment according to the 3-factor model as defined by Sudbury-Riley et al [16]: “awareness of internet health resources” (items 1 and 2), “skills and behavior needed to access health information” (items 3-5), and “self-belief in ability to evaluate health resources” (items 6-8). This domain-specific approach allows for a more nuanced evaluation of patients’ competencies in accessing, using, and appraising online health information rather than relying solely on a total eHEALS score [16].

The second section of the questionnaire included questions on self-reported sociodemographic factors and health issues and was developed based on known risk factors associated with low digital health literacy in patients with cardiac disease [17-19]. Sociodemographic factors included age, gender, educational level, cohabitation status, and social support for technology use. Patients were also asked to specify any health issues beyond the cardiac disease. Educational levels were categorized according to the International Standard Classification of Education as high school (0-2 years of post–high school education), undergraduate (3-4 years of post–high school education), and postgraduate (>4 years of post–high school education) [20]. Cohabitation status was determined as either being a cohabitant or living alone. Social support for technology use was defined as the availability of help from friends or family in addressing technological challenges when necessary. Health issues beyond the primary cardiac diagnosis were represented by a checklist of the most common health issues, including diabetes, pulmonary disease, vascular disease, back and muscle pain, asthma, rheumatoid arthritis, osteoporosis, migraine or severe headache, and chronic kidney disease [21]. Patients could specify any additional diseases in a free-text field. Health issues were categorized as “none,” “one,” or “two or more.”

Data were collected and managed using REDCap (Research Electronic Data Capture; Vanderbilt University), hosted by Aarhus University [22].

### Statistical Methods

Patients with missing items on the eHEALS were excluded to prevent underestimation of the score. On the basis of prior studies, a nonnormal distribution of eHEALS scores was anticipated, and therefore, nonparametric tests were expected to be appropriate. Normality of the data was visually assessed before selecting the statistical approach. Log transformation was attempted but did not normalize the distribution; thus, nonparametric tests were applied.

We assessed differences in eHEALS scores using the Mann-Whitney *U* test for dichotomous variables and the Kruskal-Wallis test for categorical variables with 3 levels. The correlation between age and eHEALS scores was evaluated using the Spearman rank correlation, with values interpreted as weak (0.10‐0.30), moderate (0.31‐0.50), or strong (>0.50), consistent with commonly accepted conventions in the literature [23]. The 3 subscales of the digital health literacy assessment were analyzed by calculating medians and IQRs for each subscale.

All analyses were performed using the R statistical software (version 4.1.2; R Foundation for Statistical Computing) and RStudio (version 2024.12.0; Posit PBC).

### Ethical Considerations

The questionnaire included a preamble outlining the study’s objectives, the voluntary nature of participation, and the confidentiality and anonymity of respondents. Participants were informed that no personally identifiable data (eg, name, civil registration number, or contact information) were collected. All data were collected and stored in a secure environment accessible only to members of the research team and handled in accordance with the General Data Protection Regulation and institutional data protection policies. Data were analyzed and reported in aggregate form only, ensuring that individual participants could not be identified. Informed consent was implied through completion and submission of the questionnaire. Participants were informed that they could discontinue participation at any time before submission without any consequences and that, due to the anonymous nature of the survey, withdrawal after submission would not be possible. No financial or other compensation was provided for participation. This study was conducted in accordance with the Declaration of Helsinki. The Research Ethics Committee of the Danish Regions waived the need for study approval as the study was exempt from registration under Danish law (Danish Committee Act on Research Ethics Review of Health Research Projects §14 subsection 2; case number: 2400348) [24].

## Results

### General Characteristics

In total, 54.3% (313/576) of the eligible respondents were included. Separate response rates among eligible patients at the 3 sites were 71.1% (133/187), 58.5% (134/229), and 28.8% (46/160). Baseline characteristics of the study population are summarized in Table 1.

**Table 1.** Sociodemographic characteristics and eHealth Literacy Scale (eHEALS) score of study participants (n=313).

Participant characteristic	Values
Sociodemographics	
Age (y), mean (SD)	65.8 (11)
Gender (missing: 2; n=311), n (%)	
Male	232 (74.6)
Female	79 (25.4)
Educational level (missing: 8; n=305), n (%)	
High school	92 (30.2)
Undergraduate	170 (55.7)
Postgraduate	43 (14.1)
Cohabitation status (missing: 0), n (%)	
Living alone	78 (24.9)
Cohabiting	235 (75.1)
Close relationships (missing: 3; n=310), n (%)	
Reported close personal relationships	276 (89.0)
No close personal relationships	34 (11.0)
Concurrent health issues (missing: 16; n=297), n (%)	
Number of health issues	
None	135 (45.5)
1	102 (34.3)
≥2	60 (20.2)
Digital health literacy level, median (IQR)	
eHEALS score (8-40)	30 (27-32)

**Figure 1.**
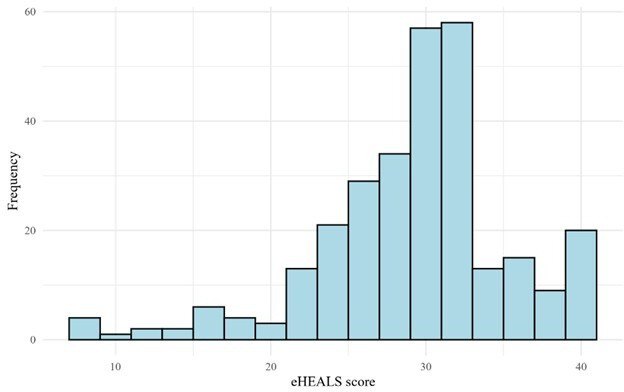
Distribution of total eHealth Literacy Scale (eHEALS) scores among participants (n=291)*.*

**Figure 2.**
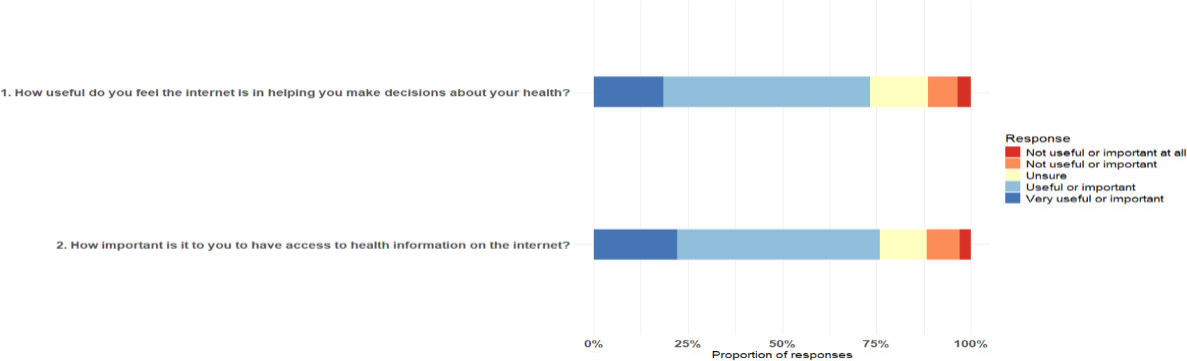
Distribution of responses to the 2 questions supplementing the eHealth Literacy Scale on the usefulness and importance of online health information.

As anticipated, eHEALS data were not normally distributed (Figure 1). The median eHEALS score was 30 (IQR 27‐32), and 91.8% (267/291) of the participants scored 26 or higher, indicating that participants generally possessed high self-reported digital health literacy levels. A total of 7% (22/313) of the questionnaires were discarded due to missing items. As a complement to the eHEALS, patients were asked about their opinions on the importance and usefulness of digital health. Just over half of the participants rated the internet as “useful” for health-related decisions (160/291, 55%) and considered access to online health information to be “important” (157/291, 54%) to them (Figure 2).

**Figure 3.**
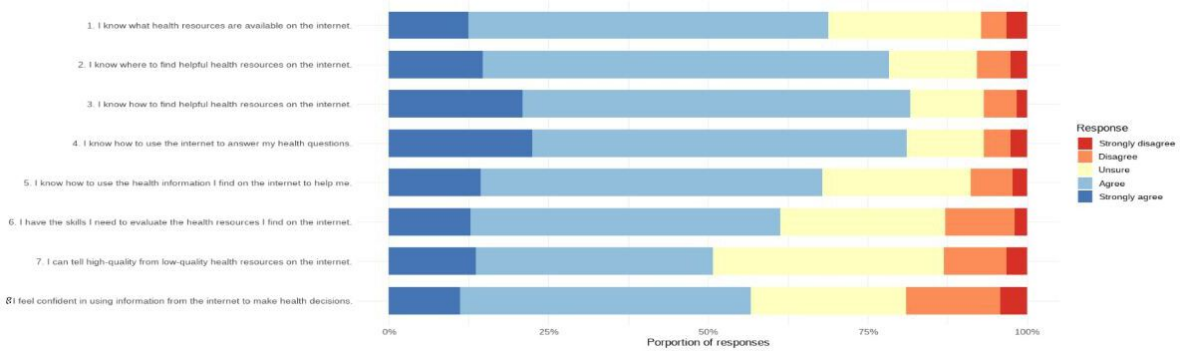
Distribution of eHealth Literacy Scale responses grouped according to the 3-factor model: “awareness” (items 1-2), “skills” (items 3-5), and “evaluate” (items 6-8).

The 3 subscales of the digital health literacy assessment were analyzed separately. The median scores were 8 (IQR 8-8) for “awareness” (2 items; maximum score=10), 12 (IQR 10-13) for “skills” (3 items; maximum score=15), and 12 (IQR 10-12) for “evaluate” (3 items; maximum score=15). These results indicate similar levels across all 3 domains. The proportion of responses across the 5-point Likert scale for each question is illustrated in Figure 3. Across all 8 eHEALS items, most patients selected “agree,” indicating an overall high perceived digital health literacy. Response distributions across items suggest lower confidence in the “evaluate” domain. Notably, for item 7 (“I can tell high-quality from low-quality health resources on the Internet”), a substantial proportion of participants responded with “unsure,” indicating limited confidence in critically appraising online health information.

### Sociodemographic Factors, Health Issues, and eHEALS Scores

Most participants (276/310, 89.0%) reported having close relationships that could assist them with technical issues. The most common sources of social support were children and spouses.

Age was significantly negatively correlated with eHEALS scores (Spearman ρ=–0.18; *P*=.002), reflecting a weak decline in self-reported digital health literacy as age increases. Additionally, a significant difference in eHEALS scores was observed across educational levels, with higher educational level corresponding to higher eHEALS scores (Kruskal-Wallis test: *χ*^2^_2_=17.0; *P*<.001).

No statistically significant differences in eHEALS scores were found across gender, cohabitation status, presence of close relationships, or number of health issues. The results are presented in Table 2 and as box plots and scatterplots in Multimedia Appendix 1.

**Table 2.** Impact of sociodemographic factors and health issues on self-reported digital health literacy level.

Variables	eHEALS^a^ score (8-40), median (IQR)	Test statistic	*P* value
Age (y)	30 (27-32)	–0.18^b^	.002
Gender		7426.5^c^	.46
Male	30 (26-32)		
Female	30 (29-32)		
Educational level		17.0^d^	<.001
High school	29 (25-32)		
Undergraduate	31 (27-32)		
Postgraduate	33 (30-39)		
Cohabitation status		8317.5^c^	.56
Living alone	30 (27-32)		
Cohabiting	31 (27-32)		
Close relationships		3992.5^c^	.47
Yes	30 (27-32)		
No	30 (23-32)		
Health issues		0.1^d^	.94
None	31 (27-32)		
1	30 (27-32)		
≥2	30 (26-34)		

a eHEALS: eHealth Literacy Scale.

b Spearman rank correlation.

c Mann-Whitney *U* test (*W* statistic).

d Kruskal-Wallis test (chi-square value with *df* of 2)*.*

## Discussion

### Principal Findings

In this cross-sectional study of patients referred to elective open-heart surgery, we found that self-reported digital health literacy was generally high, with younger age and higher educational level associated with higher eHEALS scores. No significant differences were observed for gender, cohabitation status, presence of close relationships, or number of concurrent health issues. Despite high overall eHEALS scores, many patients reported limited confidence in their ability to critically appraise online health information. To our knowledge, this is the first assessment of self-reported digital health literacy in a preoperative cardiac surgery population. Evaluating digital competencies is particularly important in this high-risk group, which faces existential threats and complex care demands. Here, digital health literacy is essential to support informed decision-making, perioperative care, and postoperative cardiac rehabilitation.

Our finding related to younger age and higher self-reported digital health literacy is consistent with prior evidence from a systematic review of 17 studies in the general population [25], as well as a cross-sectional study of individuals with moderate to high cardiovascular risk [17]. While some studies have not found an association between age and self-reported digital health literacy measured using the eHEALS, their generalizability is limited due to factors such as small, selective samples, as is the case in 2 Danish studies on patients with cardiac disease [19,26]; selection bias from motivated participants with cardiac disease in a randomized controlled trial [27]; and limited applicability to the European context of an Asian cohort from the general population [28]. Recent evidence shows a general trend toward narrowing age-related gaps in digital health literacy [9,10,18], likely due to increased digitalization and technology adoption among older adults. Our findings indicate that age-related disparities in digital health literacy persist even in Denmark, a highly digitalized country where approximately 90% of older adults use the internet and own smartphones [29]. This potential among older adults underscores the ongoing need to provide adequate support to this group to ensure that they benefit from digital health solutions [27,30].

Consistent with previous research [17,25], patients with higher educational levels reported high digital health literacy levels measured using the eHEALS. However, patients with lower educational levels also demonstrated moderately high scores, indicating less disparity in digital health competencies within this population than anticipated.

No association was found between the number of health issues and eHEALS scores. Although direct evidence is lacking, it is plausible that repeated engagement with digital health services in a highly digitalized system may help maintain or enhance digital health literacy among patients with multiple chronic conditions, potentially counteracting any negative effects of higher disease burden [31].

The findings highlight that, although certain sociodemographic factors may influence digital competencies, they do not fully explain individual variation in digital health literacy. In a health care context in which digital solutions are increasingly integrated into perioperative and rehabilitative care, awareness of these individual differences remains essential. Tailoring digital health solutions to patients’ varying levels of digital competence can enhance accessibility, promote equity, and help prevent the deepening of existing health disparities [32].

Assessing digital health literacy in cardiac surgery patients is important when identifying those who may require support to effectively engage with digital health information during the preoperative and rehabilitation phases. The eHEALS was selected for its validated, concise format, which allows for practical screening in the stressful, time-constrained preoperative setting. Although it provides only a focused assessment of patients’ ability to seek, find, and appraise online health information, it remains suitable for identifying patients who might benefit from additional guidance. More comprehensive instruments such as the Digital Health Literacy Instrument [33] or the eHealth Literacy Questionnaire [34] offer broader assessments but are less practical for routine clinical use.

The widely used eHEALS provides a brief assessment of perceived digital health literacy, but as it was developed in 2006, it does not reflect the competencies needed to engage with modern digital health technologies. Specifically, it does not address users’ ability to interpret and use data from mobile health apps and wearables, evaluate the credibility of health information on social media and video-sharing platforms, or navigate secure patient portals to communicate with health care providers. Furthermore, it lacks items related to privacy, data security, and critical appraisal of digital sources. Therefore, high eHEALS scores may primarily reflect proficiency in health 1.0 skills (eg, searching for health information online) while masking potential gaps in the more interactive and participatory health 2.0 skills required in contemporary digital health ecosystems [35]. This highlights the need for updated instruments that capture these multidimensional competencies and remain practical for use in clinical research and routine care. Screening alone cannot address digital health disparities; effective solutions also demand enhanced digital health literacy among health care providers and system-level support [36]. However, there is a marked gap between these ambitions and clinical reality. Studies indicate that older adults and individuals with limited health or digital literacy often face structural and educational barriers, which remain inadequately addressed despite the global policy focus [37,38]. Assessing digital health literacy is only the first step in identifying patients who may benefit from tailored support and in guiding the design of digital health tools that are accessible to patients with varying levels of digital competence.

Our exploratory analysis of the 3 digital health literacy subscales (“awareness,” “skills,” and “evaluate”) indicates that patients may particularly struggle with critically appraising digital health information. Such limitations may increase the risk of misinterpreting online content, potentially affecting patient-physician communication and informed decision-making [39]. The assessment of separate domains in the 3-factor structure can reveal nuances that the overall score might hide [16]. Our findings suggest that targeted support to enhance evaluation skills may help patients engage more effectively and safely with digital health information.

Participants generally stated that it was “important” for them to have access to online health information and that the internet was “useful” in helping them make decisions regarding their health. This self-reported confidence likely reflects the well-established digital health infrastructure in the Danish health care system, which promotes patient engagement and access to personal health data [40]. Such a digital infrastructure likely contributes to patients’ perceived ability to manage their health information online. This aligns with evidence suggesting that the use of internet-based health interventions can enhance digital health literacy [41].

### Strengths and Limitations

The inclusion of 3 university hospitals covering approximately two-thirds of the national population is a major strength that enhances the generalizability of our findings. However, the exclusion of patients from the Capital Region, which is characterized by a younger and more highly educated population, may result in an underestimation of overall digital health literacy and limit the completeness of national representation.

Denmark’s advanced digital infrastructure makes it a “critical case” for examining the integration of digital health solutions, offering valuable insights applicable to other countries navigating the digitalization of health care [40]. However, in settings with less integrated digital health systems or lower population-level digital competence, digital health literacy levels may be lower, and engagement with digital platforms may be more limited. Previous work has emphasized that differences in digital health literacy must be addressed to avoid inequities, highlighting the importance of simplifying communication and providing flexible solutions for disadvantaged populations [6]. The all-comer design, focusing specifically on elective open-heart surgery patients, further strengthens this study by targeting a group well positioned to benefit from tailored digital health interventions during recovery.

This study also had several limitations. First, the representativeness of the sample may be limited. Overall response rates were modest across all 3 sites, with particularly low participation at one site. Staff involved in questionnaire distribution reported that nonresponse was largely random due to logistical challenges, with only a minority attributable to patient exclusions or refusals. For context, data from the Western Denmark Heart Registry, which mandatorily records all cardiac surgery procedures in the catchment areas of the 3 participating regions, show that, for the same year, the mean age (65.9, SD 10.6 years) and proportion of male individuals (76.9%) were nearly identical to those of our sample (mean age 65.8, SD 11 years; 232/311, 74.6%), supporting reasonable representativeness. However, selection bias cannot be entirely excluded. Excluding 22 patients with incomplete eHEALS questionnaires from the analysis may also have introduced selection bias.

Second, the questionnaire was administered on the day immediately preceding major surgery. Preoperative stress and anxiety could have influenced patients’ self-reported digital health literacy, potentially inflating scores through social desirability or deflating them due to impaired concentration. However, as patients routinely receive electronic preoperative information, this timing mirrors real-world clinical practice.

Third, acute and subacute patients were not included; while their baseline digital health literacy remains unclear, their capacity to engage with digital health tools may be temporarily compromised due to the urgency of their conditions.

Finally, the use of the self-reported eHEALS, which does not fully capture the rapidly evolving digital health environment, may lead to an overestimation of patients’ true digital health literacy levels.

### Conclusions

This study demonstrates that patients scheduled for elective open-heart surgery generally report high digital health literacy, particularly younger individuals and those with higher levels of education. However, variability exists across sociodemographic groups, and challenges remain in patients’ ability to critically evaluate online health information. The findings highlight the importance of health care professionals being aware of patients’ varying levels of digital health literacy to provide appropriate support. Tailoring digital interventions based on patients’ capabilities is essential to ensure equitable access and optimize the integration of digital health solutions into cardiac care.

## Supplementary material

10.2196/83454 Multimedia Appendix 1 Box plots and scatterplot presenting the distribution of eHealth Literacy Scale scores across gender, age, presence of close relationships, educational levels, cohabitation status, and number of health issues.

## Abbreviations

eHEALS: eHealth Literacy Scale
REDCap: Research Electronic Data Capture
STROBE: Strengthening the Reporting of Observational Studies in Epidemiology
