# Supplementary material for: Digital Health Literacy in Elective Open-Heart Surgery Patients: Cross-Sectional Study
Source: JMIR Cardio. 2026 Feb 27;10:e83454. doi: 10.2196/83454 (PMC12954704; doi:10.2196/83454)
Supplement: Multimedia Appendix 1 [file cardio-v10-e83454-s001.docx]

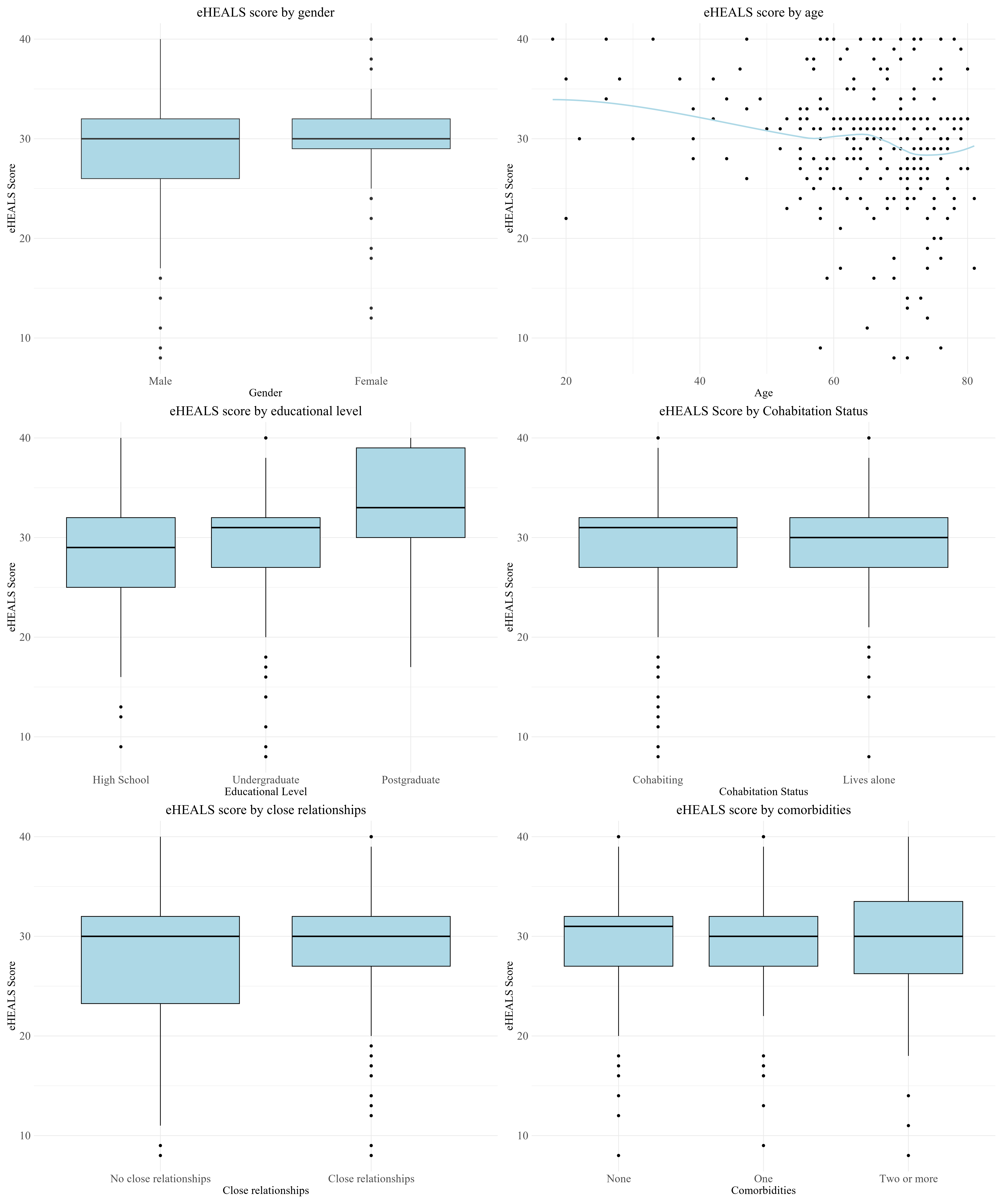


***Boxplots and scatterplot*** *presenting the distribution of eHEALS score across genders, age, relationships, educational levels, cohabitation status and number of comorbidities.*
